# Influence of the Time of Day on Triage-to-Nerve Block Duration in Acute Hip Fracture Patients

**DOI:** 10.7759/cureus.72531

**Published:** 2024-10-28

**Authors:** Bevan Jay, Tatiana Lykina

**Affiliations:** 1 Emergency, Oceania University of Medicine, Apia, WSM; 2 Allergy and Immunology, Oceania University of Medicine, Apia, WSM

**Keywords:** anterior iliac block, emergency department analgesia, fracture around hip, hip analgesia, hips, lateral femoral cutaneous nerve block, nerve block, non-surgical hip pain treatment, psoas compartment block, triage

## Abstract

Purpose: To determine whether the time of day a patient presents to the emergency department with a hip fracture affects the speed at which a nerve block is administered for analgesia. This study hypothesizes that patients presenting outside of regular hours (night shift) may experience longer delays in receiving nerve block for analgesia.

Methods: This is a single-site, retrospective, comparative, observational study of all patients triaged at an emergency department and diagnosed with a hip fracture in the 2023 calendar year. The study compared the time taken from triage to when the patient received a nerve block for analgesia between day and night shifts. The median time taken was compared using the Mann-Whitney U test.

Results: The results of 56 patients (30 day shift and 26 night shift) were compared. The median time to nerve block was four hours and 46 minutes for the day shift and five hours and 51 minutes for the night shift. This difference of one hour and five minutes did not meet statistical significance (p = 0.0556).

Conclusion: The Mann-Whitney U test indicated that the difference in time to administer a nerve block between the day and night shifts is not statistically significant at the 0.05 significance level.

## Introduction

Approximately 19,000 people over the age of 50 years are hospitalized with a hip fracture each year in Australia, the majority of which occur in patients over 65 years and result from low-energy falls [1]. This injury is associated with high mortality [2]. For patients who survive this injury, morbidity is also high, with as many as one in three elderly survivors reaching their pre-injury independence levels [3].

A report from the UK demonstrated that 31% of patients with a fractured neck of the femur were in severe pain in emergency departments (ED) with another 33% of patients experiencing moderate pain [4]. Pain is often undertreated in elderly patients, which is not only inhumane but increases the risk of chronic pain syndrome, length of stay in hospital, delirium, and prolonged recovery [5]. Optimizing pain relief for these patients is advocated to increase mobility and restrict functional loss [6]. Suitable analgesia can specifically reduce the incidence of delirium and its associated complications [7]. Nerve blocks are an important part of the pain management of these patients [8] as they provide superior analgesia compared to no nerve block or standard care [9]. Nerve blocks for this patient cohort can also reduce the risk of confusion, chest infection, and time to first mobilization [10] and increase hip flection, allowing them to sit more comfortably while in hospital [11] as well as shorten the number of days that a patient is required to stay in the hospital [12,13]. Concerningly, minimal amounts of analgesia and lengthy time to first analgesia (median 75 minutes) have been documented in Australian emergency departments, with many patients not having any recorded analgesia administration in the ED [14].

The Hip Fracture Clinical Care Standard (September 2016) by the Australian Commission on Safety and Quality in Health Care was produced with the goal to "improve the assessment and management of patients with a hip fracture to optimize outcomes and reduce their risk of another fracture" [15]. Quality statement 2a within this clinical standard is concerned with pain management and states that a patient with a hip fracture receives pain management, including the use of multimodal analgesia, if clinically appropriate. Multimodal analgesia involves the selective use of specific drugs in combination. The concept relies on using multiple analgesic drugs with different modes of action (for example, non-opioid combined with an opioid) or by different routes of administration (for example, local anesthetic block combined with a systemic analgesic). The policy in the hospital where research was conducted involves considering femoral nerve block or fascia iliac block if systemic analgesia is inadequate or to limit opioid dosage. This is in addition to regular simple analgesia.

In a retrospective descriptive cohort study in Emergency Medicine Australasia, it was demonstrated that there was a significant relationship between the access block occupancy quartile ("busier" 25% of the time) at the time of patient presentation to an emergency department and the time taken for patients to receive definitive surgical care for a neck of the femur fracture [16]. Whether analgesia administration is also delayed under similar circumstances was not investigated. When different eight-hour shifts were categorized into lower and higher quartiles of overcrowding in emergency departments, overall mortality for all patient presentations increased for the busier periods at 10 days [17]. The combination of these considerations suggests the possibility of time delays and poor outcomes for patients who present to the ED with hip fractures at times of emergency department overcrowding.

A 2006 paper from the Journal of the American Geriatrics Society investigated the effect of emergency department crowding specifically on the management of pain in adults with hip fractures. This article concluded that “factors intrinsic to the ED environment that may be associated with ED crowding, it may be possible to develop models for future prospective studies and target areas of quality improvement” [18]. One such model was proposed that measured various input, throughput, and output factors, and accounted for 31.5% of variability in mean daily length of stay in emergency departments [19]. The proposed study expands on this idea, with ED overcrowding fluctuation between day and night shifts being the quality improvement target area of investigation.

Furthermore, a consensus conference from 2011 Academic Emergency Medicine highlighted “What metrics should be used to determine ED safety?” as one of the seven research priorities for maintaining safety in the crowded ED [20]. The time of day in general has been implicated in prolonged wait times in the wider cohort of emergency department patients [21]. This research proposes investigating time to analgesia (specifically, nerve blocks) as one of these metrics, just as similar times have been investigated for other conditions such as strokes [22]. If a discrepancy between average times is found, then this could guide policy changes to account for this factor when patients with hip fractures present to emergency departments at times of overcrowding, thus improving outcomes for this patient cohort.

The purpose of this study is to determine if the speed at which a patient presenting to the emergency with hip fractures is given a nerve block for analgesia is impacted by the time of day the presentation occurred. We hypothesized that patients who present with this kind of injury outside the times of 0800 to 1800 may experience longer wait times to receive a femoral nerve block to provide analgesia for their traumatic injuries. The null hypothesis will be met if there is no statistically significant difference in the median triage to nerve block time interval between these groups. Hip fractures for the purpose of this study include intracapsular (femoral neck and head) and extracapsular (intertrochanteric and subtrochanteric) fractures.

## Materials and methods

Data were collected from a chart review of eligible patients (patients who attended the selected emergency department within a 12-month period who were diagnosed with the neck of the femur or hip fracture). The 12-month period was 01/01/2023 to 31/12/2023. The hospital where this research was performed (Peel Health Campus) has an electronic coding system for emergency department presentations post diagnosis. This system was used to generate a list of patient charts to review based on the diagnosis of hip fracture. This retrospective study had a target sample size of 50. Data collected from charts included the time/date of triage, age of the patient, nerve block performed (yes/no), and time/date when the nerve block was performed. Results were separated into patients who were triaged between the hours of 0800 and 1800 and outside these times. Patients under the age of 50 years were excluded from the analysis. If no documented evidence of nerve block was found, these patients were also excluded from the analysis.

Statistical analysis

To determine the sample size required for our study, we conducted a power calculation based on the Mann-Whitney U test. We set the confidence level to 95% and aimed for a power of 80%. The anticipated difference between median 1 and median 2 was 60 minutes, with a population standard deviation of 90 minutes. Our analysis projected a minimum sample size of 41 participants who received nerve blocks to achieve sufficient statistical power. Ethics approval, including a consent waiver, and review of the statistical analysis plan was obtained from Ramsay Health Care, Ramsay Health Care WA/SA Human Research Ethics Committee (2024/ETH/0006) prior to the commencement of data collection. All data extraction was performed on-site by the primary researcher.

The analysis plan on data collected included calculating the median time of triage to the nerve block of both groups (triaged between 0800 and 1800 and triaged between 1800 and 0800). A sum of ranks was calculated for each data set. We applied the Mann-Whitney U test to compare continuous data (e.g., triage to nerve block times) between day and night shifts. The test assumes that the two groups being compared are independent, meaning that the data points in one group do not influence the data points in the other. Additionally, the Mann-Whitney U test is appropriate for continuous data as it ranks the values between groups rather than relying on distributional assumptions of normality. This test was chosen to account for the potential non-normal distribution of the data while allowing for a meaningful comparison of the two independent groups. The alpha level was set at 0.05.

## Results

**Figure 1:**
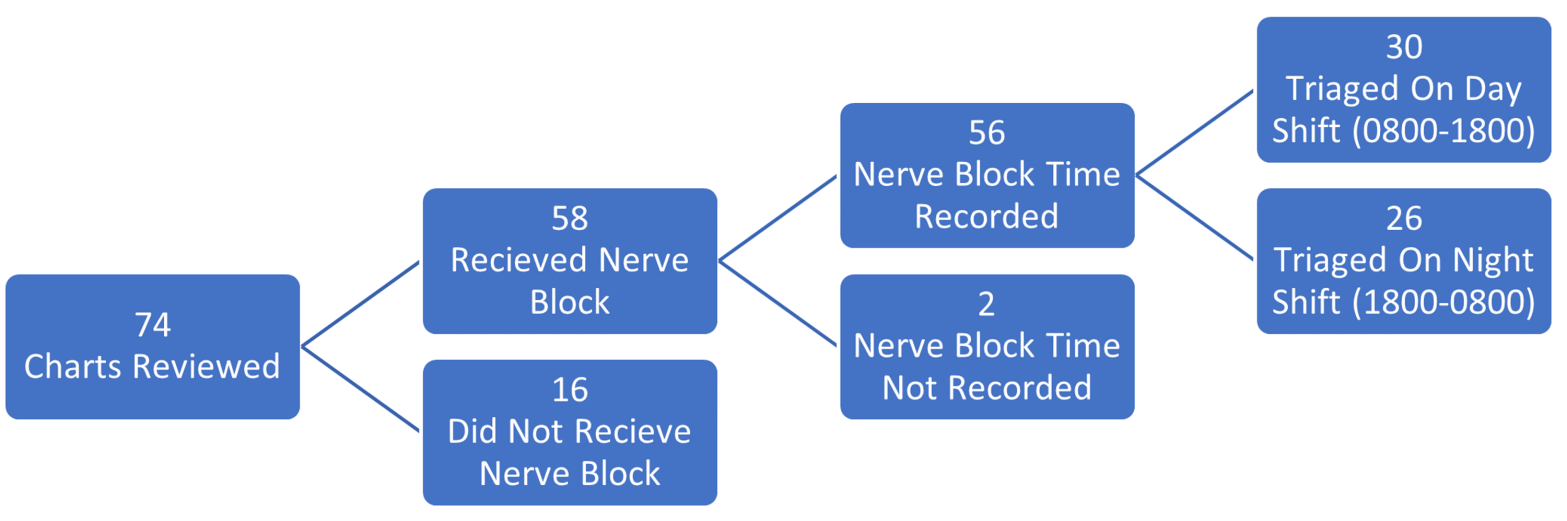
Patient inclusion.

A total of 74 charts were reviewed. Sixteen patients did not receive a nerve block. Two patients received a nerve block, but no time was documented. A total of 56 patients were included in the primary outcome. Of these patients, 26 were triaged on the night shift and 30 were triaged on the day shift (Figure 1).

**Figure 2:**
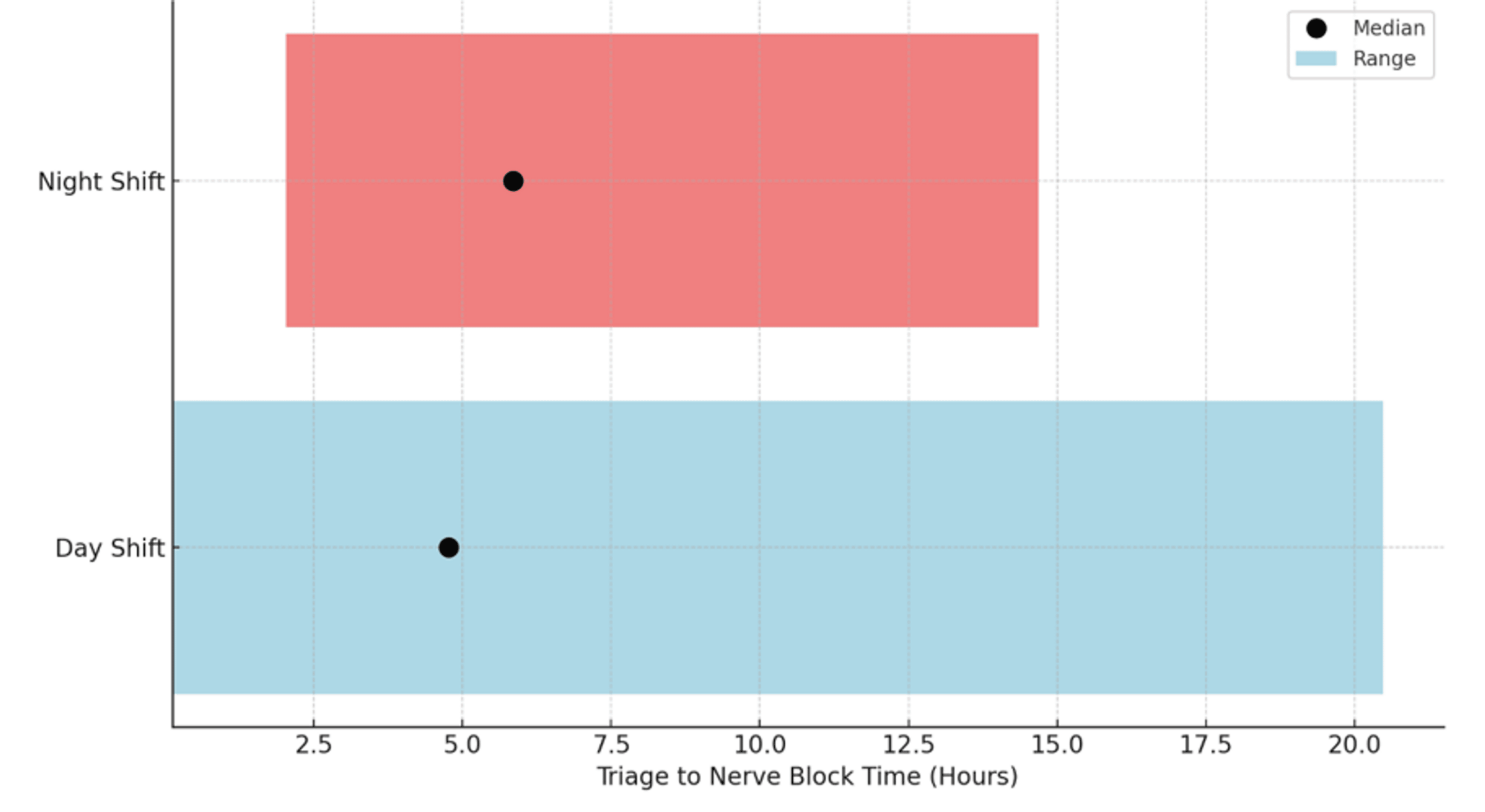
Range and median of triage to nerve block time by shift.

For the day shift group (0800-1800), the range of triage to nerve block time was from seven minutes to 20 hours and 29 minutes, with a median of four hours and 46 minutes. For the night shift group (1800-0800), the range of triage to nerve block time was from two hours and two minutes to 14 hours and 41 minutes, with a median of five hours and 51 minutes (Figure 2). This represented a one hour and five minutes longer median time in the night shift group.

The Mann-Whitney U test was conducted to compare the time to administer a nerve block in the emergency department during the day and at night (Table 1). To assess statistical significance, the U statistic was used to compare the critical value from the Mann-Whitney U distribution table for the day group (n = 30) and the night group (n = 26) at an alpha level of α = 0.05. The critical value for a sample size of 30 and 26 was 270. The smaller U statistic (UD = 273) was higher than the critical value (270), corresponding to a p-value of 0.0556 in this study. Therefore, the difference between the day and night groups did not meet statistical significance.

**Table 1:** Primary outcome. * Hours:minutes. ** Lower critical value of 270 for a sample size of 30 and 26.

	Day group (0800-1800)	Night group (1800-0800)
Sample size (n_D_)	30	26
Median nerve block time*	4:46	5:51
Sum of ranks	738	858
U statistic (U_D_)**	273	507

## Discussion

Although the 65-minute longer median wait time for nerve block in the night group did not reach statistical significance at the alpha level of 0.05, it may still highlight the need for targeted quality improvement strategies for hip fracture pain management during the after-hours period in this emergency department. Prolonged wait time to receive a nerve block is likely to be clinically significant for individual patients. Further studies with larger patient samples across multiple sites are warranted to determine if this observed difference is a true phenomenon or if it merely reflects a localized trend. Addressing these delays could enhance patient outcomes and improve the standard of care for hip fracture management in the after-hours setting.

It is noted from the results that only seven out of 26 (27%) treated patients on the night shift received their nerve block within four hours, whereas 13 out of 30 (43%) patients did so on the day shift. These results suggest that Peel Health Campus is not meeting WEAT targets for this patient cohort, despite the time of day that they were seen. The Western Australia Emergency Access Targets from the jurisdiction in which this hospital is located require that 90% of all patients presenting to a public hospital ED in Western Australia be seen and admitted, transferred, or discharged within four hours [23]. For some time-critical presentations at triage (i.e., stroke), specific protocols have been developed to ensure that appropriate investigations and treatments are performed in a timely manner. Similar policy changes in regards to triaging patients with suspected hip fractures could improve treatment times and outcomes.

Overall, 78% (74% day shift/84% night shift) of patients with hip fractures received a nerve block, which is much higher than that reported in a retrospective single-site observational study (45%) from another regional Australian ED in 2017 [24]. It is unclear which doctors were performing these tasks (registered medical officer, registrar, or consultant) and whether further training of more junior staff could help reduce the above-mentioned times. A previous randomized controlled trial comparing anesthesiologists to specially trained nurses suggested that nurses were able to provide ultrasound-guided nerve blocks earlier with similar outcomes from the procedure [25].

Limitations

Several limitations could impact the validity and generalizability of the findings in this study.

Retrospective Design/Incomplete Data

The study relies on existing records, which may be incomplete or inaccurate, leading to potential biases or missing data. For instance, two patients were documented to have received a nerve block but no time was documented for these patients (both on the day shift). The inclusion of these results may have changed the median time if included.

Retrospective Design/Uncontrolled Confounding Variables

Retrospective studies cannot control for all potential confounding variables, such as staffing levels, severity of injury, or concurrent medical conditions, which could influence the time to nerve block. The skill mix of doctors who covered night shifts during the specified time period may not be reflective of the skill level (specifically, the ability to perform a nerve block) of doctors who covered more of these shifts in other time periods.

Small Sample Size

With a target sample size of 50, the study may be underpowered to detect significant differences, especially if the effect size is small. This could increase the risk of type II errors (failing to detect a true difference).

Selection Bias

Patients who did not receive a nerve block were excluded, which could introduce selection bias. If the decision not to perform a nerve block is related to factors such as time of presentation, this could skew the results.

Generalizability

The study is conducted at a single hospital (Peel Health Campus), which may limit the generalizability of the findings to other settings with different patient populations, staffing levels, or protocols. Unlike most metropolitan public access emergency departments, this one is owned by a private medical group rather than the state government. This study in other emergency departments could have significantly different results.

Time-Based Categorization

The study categorizes patients based on the time of triage, but other factors (e.g., time to doctor’s assessment) might also influence the timing of the nerve block. This simplification could overlook important nuances in patient care.

Potential for Documentation Errors

If there is inconsistent or inaccurate documentation in the patient charts regarding the timing of nerve blocks, this could introduce measurement bias. It is noted that the time of the nerve block was sometimes written manually into the notes and sometimes on a procedure sticker.

Exclusion of Patients Without Nerve Blocks

By excluding patients who did not receive a nerve block, the study may overlook important information about why these patients were not treated in this way. This exclusion might lead to an incomplete understanding of the factors influencing treatment decisions.

Temporal Variations in Hospital Operations

Differences in hospital operations, staffing, and patient load between daytime and nighttime could confound the relationship between time of day and nerve block timing. Without adjusting for these variations, the results might not accurately reflect the impact of time of day alone. Specifically, there are often afternoon shift doctors who are present in part of both time periods. It is unclear if the presence of these staff members impacts the results of the study.

Pain management for patients with hip fractures can vary between emergency departments and guidelines. This study contributes to the needed research and quality improvement for these patients. Further research is required to identify and assess the implementation of effective strategies to improve the prompt utilization of nerve blocks in the emergency department setting for patients in pain with hip fractures.

## Conclusions

The Mann-Whitney U test indicated that the difference in time to administer a nerve block between the day and night shifts is not statistically significant at the 0.05 significance level (p = 0.0556). Therefore, we fail to reject the null hypothesis. Although there was no statistically significant difference, a median difference of 65 minutes was observed between day and night shifts. Further research over multiple sites is required to determine if there is a more generalized difference between the time to nerve block between the day and night shifts.
